# Brown Adipocyte BMAL1 Deficiency Is Associated with PVAT Remodeling and Increased Atherosclerosis

**DOI:** 10.3390/cells15191791

**Published:** 2026-09-30

**Authors:** Ruiyan Xu, Zhenguo Wang, Wenjuan Mu, Juan Zhong, Yao Liu, Yanhong Guo, Jifeng Zhang, Y Eugene Chen, Guohua Li, Lin Chang

**Affiliations:** 1 Key Laboratory for Arteriosclerology of Hunan Province, Institute of Cardiovascular Disease, Hunan International Scientific and Technological Cooperation Base of Arteriosclerotic Disease, Department of Pathophysiology, Hengyang Medical School, University of South China, Hengyang 421001, China; lauraryan@foxmail.com (R.X.); yaoliua@umich.edu (Y.L.); 2 Department of Internal Medicine, Frankel Cardiovascular Center, University of Michigan, Ann Arbor, MI 48105, USA; zhenguow@med.umich.edu (Z.W.); wenjmu@med.umich.edu (W.M.); zhongjuan126@gmail.com (J.Z.); yanhongg@med.umich.edu (Y.G.); jifengz@med.umich.edu (J.Z.); echenum@med.umich.edu (Y.E.C.)

**Keywords:** BMAL1, ChREBP, PVAT, atherosclerosis

## Abstract

Perivascular adipose tissue (PVAT) is increasingly recognized as an active vascular regulator that influences inflammation, metabolism, and atherosclerotic progression. In healthy vessels, PVAT displays brown adipose-like features and contributes to vascular protection, whereas PVAT dysfunction is associated with adipose whitening, inflammatory activation, and enhanced plaque formation. Circadian clock genes are major regulators of tissue metabolism, yet their role in PVAT biology and atherosclerosis remains incompletely understood. Here, we investigated the function of the core circadian factor BMAL1 in brown adipocytes and PVAT using a brown adipocyte-specific BMAL1 knockout model (*Bmal1*^BAKO^) and ApoE-deficient atherosclerosis-prone mice. Transcriptomic profiling of thoracic aortic PVAT and abdominal aortic PVAT revealed that BMAL1 deficiency induces a shift in metabolic gene expression profiles characterized by upregulation of glycolytic and lipogenic gene programs, together with suppression of genes linked to thermogenic and vascular homeostasis. Mechanistically, BMAL1 loss increased *Mlxipl* (encoding ChREBP) expression, and ChREBP knockdown attenuated the glycolytic–lipogenic transcriptional response to BMAL1 depletion in cultured brown adipocytes. In parallel, BMAL1-deficient PVAT exhibited enlarged lipid droplets, increased oxidative stress-related transcriptional profile, and inflammatory remodeling accompanied by macrophage accumulation. In ApoE^−/−^ mice, brown adipocyte-specific BMAL1 deletion aggravated atherosclerotic lesion formation in both chow-fed aging mice and Western diet-fed young mice, without major changes in systemic energy balance. In conclusion, brown adipocyte BMAL1 deficiency was associated with ChREBP-linked metabolic reprogramming, loss of brown-like PVAT features, oxidative and macrophage-associated remodeling, and increased atherosclerosis.

## 1. Introduction

Atherosclerotic cardiovascular disease remains a leading cause of morbidity and mortality and is driven by dyslipidemia, vascular inflammation, endothelial dysfunction, immune-cell recruitment, and maladaptive vascular remodeling [1,2,3,4]. Perivascular adipose tissue (PVAT) is the adipose depot immediately adjacent to the adventitia of most large and medium-sized arteries. PVAT was regarded primarily as structural support for blood vessels, is now recognized as a metabolically active local regulator of vascular tone, redox balance, inflammation, and arterial-wall homeostasis through adipokines, cytokines, lipids, reactive oxygen species, and vasoactive mediators [5,6,7,8]. However, these effects depend on vascular bed, species, anatomical location, and metabolic state [9]. In the healthy state, PVAT releases anti-contractile and anti-inflammatory mediators; while metabolic stress such as obesity, aging, dyslipidemia, diabetes, and chronic inflammation can shift its secretory phenotype toward increased production of inflammatory adipokines, cytokines, and other vasoactive factors that promotes endothelial dysfunction, immune-cell accumulation, vascular remodeling, and atherosclerotic disease progression [10,11,12]. Our prior work supports a direct relationship between dysfunctional PVAT and local atherosclerotic susceptibility, including evidence that PVAT inflammatory programs and adipocyte–vascular-cell crosstalk are associated with plaque development and adverse vascular remodeling [13,14].

PVAT is heterogeneous across vascular beds and includes white, beige, and brown adipocyte populations with distinct developmental origins and metabolic functions [6,14]. Brown-like PVAT has attracted particular attention because thermogenic adipocyte programs may preserve vascular homeostasis through local metabolic buffering, anti-inflammatory signaling, and regulation of vascular redox status. In support of a protective role for thermogenic PVAT, BMP4-mediated PVAT browning was reported to induce an anti-inflammatory program and limit atherosclerosis in mice, whereas disruption of PVAT homeostasis can facilitate a proatherogenic vascular microenvironment [9,15]. Conversely, metabolic stress can promote PVAT dysfunction, characterized by adipocyte hypertrophy, lipid accumulation, mitochondrial dysfunction, oxidative stress, immune-cell recruitment, and inflammatory cytokine production. Defining the transcriptional pathways that preserve metabolically competent PVAT may therefore identify mechanisms that limit local vascular inflammation and atherogenesis [16,17]. Nevertheless, the transcriptional mechanisms that maintain the brown-adipocyte phenotype of PVAT and couple adipocyte metabolism to vascular protection remain incompletely defined.

Circadian disruption and altered clock-gene activity are associated with metabolic and inflammatory abnormalities relevant to cardiovascular disease [18]. The core circadian-clock transcription factor brain and muscle ARNT-like 1 (BMAL1) is a compelling candidate regulator of this process. BMAL1 coordinates tissue-specific rhythms in metabolism, redox regulation, inflammation, and cellular differentiation, and its vascular consequences are strongly cell-context dependent [19,20,21]. In vascular smooth muscle cells, impaired BMAL1 expression promotes atherosclerosis by enhancing smooth-muscle migration, monocyte transmigration, and oxidative stress, while clinical carotid plaques with lower BMAL1 expression were associated with neurological symptoms [22]. Conversely, myeloid-cell Bmal1 deletion reduced lesion burden in experimental atherosclerosis [23], emphasizing that BMAL1 actions in atherogenesis cannot be inferred from global loss-of-function models and must be resolved at the cellular level.

Recent studies also establish that BMAL1 is important for brown-adipocyte metabolic function. Brown adipocyte-specific Bmal1 deletion disrupts the circadian rhythm of fatty-acid utilization, impairs thermogenesis, and reduces whole-body energy expenditure, despite increased UCP1 expression [24,25]. These findings indicate that BMAL1 controls brown-adipocyte function beyond canonical thermogenic-marker expression and raise the possibility that its loss may compromise the local metabolic and paracrine functions of brown-like PVAT. Consistent with this concept, disruption of the liver clock alters circulating metabolic substrates, remodels PVAT metabolic-gene expression, and impairs PVAT-dependent endothelium-mediated vasorelaxation [26], demonstrating that circadian disruption can affect vascular function through PVAT. However, whether brown adipocyte BMAL1 directly restrains PVAT dysfunction and atherosclerosis remains insufficiently understood.

Carbohydrate-responsive element-binding protein (ChREBP; *MLXIPL*) is a glucose-responsive transcription factor that regulates glycolytic, pentose-phosphate-pathway, and de novo lipogenic gene expression in metabolic tissues [27]. Through its effects on lipid synthesis, lipolysis-related signaling, and production of bioactive lipid mediators, ChREBP can influence adipocyte insulin sensitivity and systemic lipid handling [28]. Recent work in brown adipocytes indicates that ChREBP is required for nutrient-responsive glucose metabolism and expression of lipogenic genes [29,30]. Overexpression of ChREBP in brown adipose tissue (BAT) inhibits mitochondrial, autophagic, respiratory, and thermogenic gene programs, and results in a whitening-like phenotype [31]. By contrast, recent single-cell and functional data identified ChREBP as enriched in highly thermogenic UCP1-positive brown adipocytes and suggested that it supports brown-adipocyte development and thermogenic capacity through regulation of lipid utilization [32]. These apparently divergent findings underscore the likely importance of ChREBP isoform, expression level, nutritional state, and cellular context.

A potential BMAL1–ChREBP regulatory relationship could therefore link circadian regulation to glucose-responsive lipid metabolism in brown PVAT. Disruption of this relationship may alter brown-adipocyte metabolic flexibility and contribute to PVAT dysfunction and local inflammatory or proatherogenic signaling in the adjacent vascular environment. This model is biologically plausible because dysfunctional PVAT communicates bidirectionally with endothelial cells, vascular smooth muscle cells, and infiltrating immune cells through paracrine adipokines, cytokines, lipid mediators, and oxidative signals [11]. Defining whether brown adipocyte BMAL1 regulates ChREBP-associated metabolic programs in PVAT may thus identify a local, circadian-metabolic mechanism contributing to atherosclerotic disease.

## 2. Materials and Methods

### 2.1. Mice

Brown adipocyte-specific BMAL1 knockout (*Bmal1*^BAKO^) mice [25] were generated by crossing *Bmal1*^flox/flox^ mice (Jackson Laboratory, Bar Harbor, ME, USA, strain # 007668) with *Ucp1* promoter-driven Cre mice (Jackson Laboratory, Bar Harbor, ME, USA, strain # 024670). All lines were further bred onto an ApoE^−/−^ (Jackson Laboratory, Bar Harbor, ME, USA, strain # 002052) background. Sex and age-matched littermate *Bmal1*^flox/flox^ mice or Cre recombinase mice with ApoE knockout were used as controls. Mice were housed at the University of Michigan animal facility (Ann Arbor, MI, USA) under 22 °C and a 12-h light/12-h dark cycle with free access to chow diet (No. 2918; 18% protein, 6% fat, and moderate phytoestrogen; Harlan Laboratories, Indianapolis, IN, USA) or Western diet (TD. 88137, ENVIGO, Indianapolis, IN, USA, 42% of calories from fat and 0.2% cholesterol) and tap water. To minimize potential confounding factors, mice from different genotypes were housed under identical environmental conditions and randomly distributed across cages. Measurements were conducted using standardized protocols and identical equipment settings. Zeitgeber time (ZT) was used to standardize tissue collection. All experiments were conducted at ZT2–6 except as otherwise indicated in the figure legends. The Animal Research Ethics Committee of the University of Michigan approved the study protocol for animals. No animals or data points were excluded from the analysis. Sample size for atherosclerosis study was determined by power calculations indicated that *n* = 10–12 mice per group will provide 80% power (α = 0.05) to detect a 25% difference between groups. Mice were randomly assigned to experimental groups. The experimental unit was one mouse. Secondary molecular, histological, and cell-culture experiments were exploratory and included 3–6 independent biological replicates per group, as specified in the corresponding figure legends.

### 2.2. ELISA

The collected blood was centrifuged at 3000× *g* for 10 min at 4 °C to obtain plasma. Plasma levels of tumor necrosis factor-α (TNF-α) (Invitrogen, Waltham, MA, USA, BMS607-3) and interleukin-6 (IL-6) (Invitrogen, KMC0061) were measured using enzyme-linked immunosorbent assay (ELISA) according to the manufacturer’s protocols.

### 2.3. Metabolic Cage

Whole-body metabolic parameters were assessed using an indirect calorimetry system at the University of Michigan Animal Phenotyping Core. Mice were individually housed in metabolic chambers with free access to food and water. Oxygen consumption (VO_2_), carbon dioxide production (VCO_2_), energy expenditure (EE), respiratory exchange ratio (RER), body composition and food intake were continuously recorded. The metabolic cage environment was maintained at 22–23 °C with 39–40% humidity and a background noise level of approximately 70 dB. Mice were kept under a 12-h light/12-h dark cycle. The airflow rate in each chamber was maintained at 0.42–0.45 L/min. Gas exchange measurements were sampled for 5 s every 20 min, and locomotor activity was recorded every second throughout the monitoring period. Food intake was recorded for each mouse.

### 2.4. Tissue Collection and Histology

Interscapular BAT, thoracic PVAT and gonadal WAT were freshly collected and fixed in 10% neutral buffered formalin. Tissue samples were dehydrated using gradient ethanol and were embedded in paraffin. Paraffin blocks were sectioned at 5 μm thickness by the Pathology Core at the University of Michigan. The sections were stained with hematoxylin and eosin (H&E) for morphological analysis according to our previous studies [33]. Investigators were blinded to group allocation during plaque quantification and data analysis.

### 2.5. Lipid Droplet Size Quantification

Lipid droplet size was quantified using the Adiposoft plugin (version 1.16) in ImageJ (version 1.54). Images were calibrated based on the scale bar and lipid droplets were automatically identified using Adiposoft. Automated segmentation results were manually inspected; non-lipid regions were removed and missed droplets were manually added. The area of each lipid droplet was extracted and grouped into defined size intervals. The proportion of droplets within each size category was calculated as a percentage of the total droplet count and plotted as a lipid droplet size distribution bar graph.

### 2.6. Pathological Analysis of Atherosclerotic Lesions in the Aortic Sinus

Sections of atherosclerotic lesions in the aortic sinus region were prepared as previously described [33]. In brief, formalin-fixed aortic roots were cryoprotected in 40% sucrose overnight at 4 °C, embedded in O.C.T compound (4583, Tissue-Tek, Torrance, CA, USA), and snap-frozen in liquid nitrogen. The frozen blocks were sectioned at 5 μm on a Leica Cryostat (CM3050S, Leica, Teaneck, NJ, USA), and the slides were used for Oil Red O (ORO) staining. For aortic sinus cryosections, slides were immersed in 60% 2-propanol for 20 s, stained with ORO solution for 30 min, rinsed with 60% 2-propanol for 30 s, and washed with distilled water for 20 s. Slides were mounted with Permount^®^ (17986-01, Electron Microscopy Sciences, Morgantown, PA, USA), and images were captured using a microscope (BZ-X800, KEYENCE, Itasca, IL, USA). Lipid-positive lesion areas in the aortic sinus and whole aorta were quantified using ImageJ (version 1.54) software.

For en face analysis, whole aortic trees were immersed in 60% 2-propanol for 1 min, stained with ORO solution for 30 min, rinsed with 60% 2-propanol for 5 min, and washed with distilled water. The aortas were longitudinally opened, and images were captured using SPOT™ 5.2 software with a Nikon lens (Melville, NY, USA). En face lesion areas were quantified using ImageJ (version 1.54) software.

### 2.7. Oral Glucose Tolerance (OGTT) and Insulin Tolerance Test (ITT)

OGTT was performed after an overnight fast and acclimation to the testing room with access to water. Mice were gavaged with D-(+)-Glucose (G7528-1KG, Sigma, St. Louis, MO, USA) at 2 g per kg of body weight. ITT was performed after fasting for 6 h and acclimation to the testing room with access to water. Mice were injected intraperitoneally with Humulin^®^ R U-100 (insulin human) (NDC 0002-8215-17, Eli Lilly, Indianapolis, IN, USA) 0.75 U per kg of body weight. Blood glucose levels were measured via tail-tip sampling at 0, 30, 60, 90, and 120 min using a glucometer (Contour Next EZ, Bayer, Whippany, NJ, USA).

### 2.8. RNA Extraction, qPCR and Bulk RNA-Sequencing

Total RNA was extracted from BAT, PVAT, WAT, and cells using TRIzol reagent (Invitrogen, 15596018) and further purified using the RNeasy Mini kit (74106, QIAGEN, Germantown, MD, USA), and 2 µg of total RNA was subjected to cDNA synthesis with the SuperScript^®^ III First-Strand Synthesis System (18080051, Invitrogen, Waltham, MA, USA). Next, levels of multiple mRNAs, as indicated in the corresponding figures, were analyzed by qPCR using the primers listed in the supplemental table and are expressed relative to β-actin or *Tbp*. Relative mRNA expression was determined using the 2^−ΔΔCt^ Method. The number of biological replicates is specified in the figure legends. All qPCR analyses were performed in at least two independent experimental runs, and representative results are presented. Primer sequences used in this study are listed in Supplemental Table S1.

TA-PVAT and AA-PVAT were collected at ZT4–ZT6 from 10–12-week-old female littermate control and *Bmal1*^BAKO^ mice maintained on chow diet (*n* = 3 independent biological replicates/genotype/depot). Total RNA was isolated using RNeasy Lipid Tissue Mini Kit (74804, QIAGEN, Germantown, MD, USA). RNA concentration and purity were determined using a NanoDrop spectrophotometer, and RNA integrity was assessed using an Agilent Bioanalyzer (Santa Clara, CA, USA) before library preparation. All samples had RNA integrity numbers (RIN) > 7. RNA libraries were prepared using the NEBNext Ultra RNA Library Prep Kit (NEB #E7490, Ipswich, MA, USA) for Illumina with poly(A) selection and subjected to 151-bp paired-end sequencing on an Illumina NovaSeq platform (San Diego, CA, USA), generating an average of approximately 40 million paired reads per sample. Bcl2fastq2 Conversion Software v2.20 was used for demultiplexing and FASTQ file generation. Reads were trimmed using Cutadapt v2.3 and assessed for sequencing quality using FastQC v0.11.8. Reads were aligned to the mouse reference genome GRCm38 (ENSEMBL) using STAR v2.7.8a, and gene-level abundance estimates were generated using RSEM v1.3.3. Alignment parameters followed ENCODE RNA-seq standards, and quality-control metrics from different stages of the pipeline were aggregated using MultiQC v1.7. Differential expression was analyzed separately for TA-PVAT and AA-PVAT using DESeq2 (v1.48.1) in R (v4.5.1) with genotype as the modeled variable. Genes with fewer than 10 total counts across all samples were excluded, and remaining counts were normalized using the DESeq2 median-of-ratios method. Differentially expressed genes (DEGs) were defined by Benjamini–Hochberg FDR-adjusted *p* < 0.05 and absolute log2 fold change ≥ 1. Functional enrichment analysis was performed using Database for Annotation, Visualization, and Integrated Discovery (DAVID, v2025_2), using all retained genes as the background universe. The complete RNA-seq dataset is available in the NCBI Gene Expression Omnibus (GEO) under accession number GSE347651. Quantitative PCR (qPCR) was used to validate RNA-seq results and assess the expression of genes involved in glycolysis, lipogenesis, and BMAL1-dependent regulation.

### 2.9. Brown Preadipocyte Isolation and Differentiation

Interscapular BAT was dissected from *Bmal1*^BAKO^ or control mice. The tissues were minced into 2~3 mm pieces, digested with 1 mg/mL of collagenase P 11213865001, (Sigma, St. Louis, MO, USA) at 37 °C for 30 min, the digestion was stopped by gently pipetting the suspension a few times and adding culture medium. The digested tissues were filtered through 100 μm nylon filters (Falcon 352360, Sigma, St. Loius, MO, USA) into 15 mL conical tubes followed by centrifugation at 400× *g* for 10 min. The resulting pellet was resuspended in Dulbecco’s Modified Eagle Medium/Nutrient Mixture F-12 containing 20% fetal bovine serum and 1% pen strep and plated for further culture. All culture media and differentiation reagents were sterilized using a 0.22 μm filter (Millpore Sigma SLGVM33RS, Burlington, MA, USA).

After reaching 70% confluence, preadipocytes were transfected with lentiviral vectors for 12 h, lenti-*Bmal1* (GFP-tagged, LVS (VB9001935912nwm)-K1, VectorBulider, Chicago, IL, USA) or lenti-*ChREBP* (GFP-tagged, LVS (VB9001846485ken)-K1, VectorBulider, Chicago, IL, USA) or control lenti-*GFP* (LVM (VB0100009526zpu)-K1, VectorBulider, Chicago, IL, USA). After reaching confluence in differentiation medium (Dulbecco’s Modified Eagle Medium/Nutrient Mixture F-12 containing 10% fetal bovine serum, 20 nM insulin and 1 nM 3,3′,5-triiodo-L-thyronine), preadipocytes were induced by 0.5 mM isobutylmethylxanthine, 0.5 mM dexamethasone, and 0.125 mM indomethacin for 48 h. Next, the cells were differentiated in differentiation medium for an additional 1 week.

### 2.10. Gene Knockdown

SVF cells isolated from interscapular BAT were transduced with lentivirus encoding shRNA targeting *Bmal1* (*GFP*-tagged, LVS(VB900201-5228zvs)-K1, VectorBulider, Chicago, IL, USA) or ChREBP (*GFP*-tagged, LVS(VB900184-6485ken)-K1, VectorBulider, Chicago, IL, USA) at a multiplicity of infection (MOI) of 20 for 12 h. MOI optimization was performed prior to the experiments.

### 2.11. Chromatin Immunoprecipitation (ChIP) Assay

Mouse interscapular brown preadipocytes were isolated as described previously. Preadipocytes were transfected with lentiviral vectors as described above. Lenti-flag-tagged *Bmal1* or control lenti-*GFP* (LVM (VB0100009526zpu)-K1, VectorBulider, Chicago, IL, USA). The cells were fixed and ChIP assays were performed using SimpleChIP^®^ Enzymatic Chromatin IP Kit (9003, Cell Signalling Technology, Danvers, MA, USA). The size of the sonicated DNA fragments subjected to immunoprecipitation was 150 to 900 bp as determined by ethidium bromide gel electrophoresis. Purified chromatin was immunoprecipitated using anti-Flag monoclonal antibody (CST, 14793S) and Normal Rabbit IgG (2729S, Cell Signalling Technology, Danvers, MA, USA). Eluted DNA fragments were purified to serve as templates for the PCR amplification. The input fraction corresponded to 2% of the chromatin solution before immunoprecipitation. Primers used to amplify the area containing the E-Box binding site (1737 to 1916 bp upstream of the transcription start site) of the ChREBP gene in the ChIP assay are: forward primer 5′-CACCCGGTCTACAGTTTGGG-3′ and reverse primer 5′-CTCCTTAGCACGTGGATGGC-3′, resulting in a 180 bp fragment.

### 2.12. Immunohistochemistry

Paraffin-embedded tissue sections were deparaffinized in xylene and rehydrated through a graded ethanol series (100%, 95%, 85%, and 75%) to distilled water. Antigen retrieval was performed by incubating the sections in sodium citrate buffer (pH 6.0) using a microwave oven or water bath, followed by natural cooling to room temperature and washing with PBS three times for 5 min each. Endogenous peroxidase activity was blocked by incubating the sections with 3% hydrogen peroxide for 10 min at room temperature. After washing with PBS, sections were blocked with 5% donkey serum for 1 h at room temperature to reduce nonspecific binding. The sections were then incubated overnight at 4 °C in a humidified chamber with anti-CD68 primary antibody (PA5-109344, ThermoFisher Scientific, Waltham, MA, USA) or species-matched IgG (Cell Signaling Technology, Danvers, MA, USA, 2729S) as a negative control. The following day, sections were brought to room temperature and washed three times with PBS for 5 min each, followed by incubation with HRP-conjugated secondary antibody (ab64261, Abcam, Waltham, MA, USA) for 1 h at room temperature. After washing, color development was performed using DAB substrate solution (Abcam, ab64261), and the reaction time was monitored under a microscope before being terminated with distilled water. Sections were then counterstained with hematoxylin, rinsed under running water, dehydrated through graded ethanol, cleared in xylene, and mounted with neutral resin. Images were acquired using an Olympus DP73 microscope under identical exposure settings, and macrophage infiltration was evaluated qualitatively based on the presence of brown CD68-positive staining.

### 2.13. Statistical Analysis

Data are presented as mean ± SEM. Statistical significance was determined by two-tailed unpaired Student’s *t*-tests, Mann–Whitney tests, two-way ANOVA, repeated-measures two-way ANOVA, or mixed-effects models as appropriate. For ANOVA models involving multiple comparisons, appropriate post-hoc multiple-comparisons tests were applied as specified below. OGTT and ITT data were analyzed using repeated-measures two-way ANOVA with genotype, time, and genotype-by-time interaction, followed by Šídák’s multiple-comparisons test where applicable. Metabolic-cage data were analyzed using mixed-effects models with mouse as a random effect and genotype, time/light phase, and their interaction as fixed effects; post-hoc multiple comparisons were adjusted using Šídák’s method where applicable. Energy expenditure was additionally assessed using body mass or lean mass as a covariate where appropriate rather than ratio normalization alone. ZT expression studies were analyzed using two-way ANOVA with genotype, ZT, and their interaction, followed by Šídák’s multiple-comparisons test where applicable, because different mice were euthanized at each ZT. Lipid-droplet distributions were analyzed at the mouse level. Droplets were treated as observations nested within animals, using per-mouse bin percentages followed by two-way ANOVA with appropriate multiple-comparisons adjustment. Individual droplets were not treated as independent biological replicates. Simple two-group comparisons were performed using two-sided unpaired *t*-tests when assumptions were satisfied; otherwise, Mann–Whitney tests were used. A *p*-value < 0.05 was considered statistically significant. Statistical significance is denoted as *p* < 0.05 (*) and *p* < 0.01 (**).

## 3. Results

### 3.1. BMAL1 Deficiency Altered PVAT Transcriptional Programs

**Figure 1:**
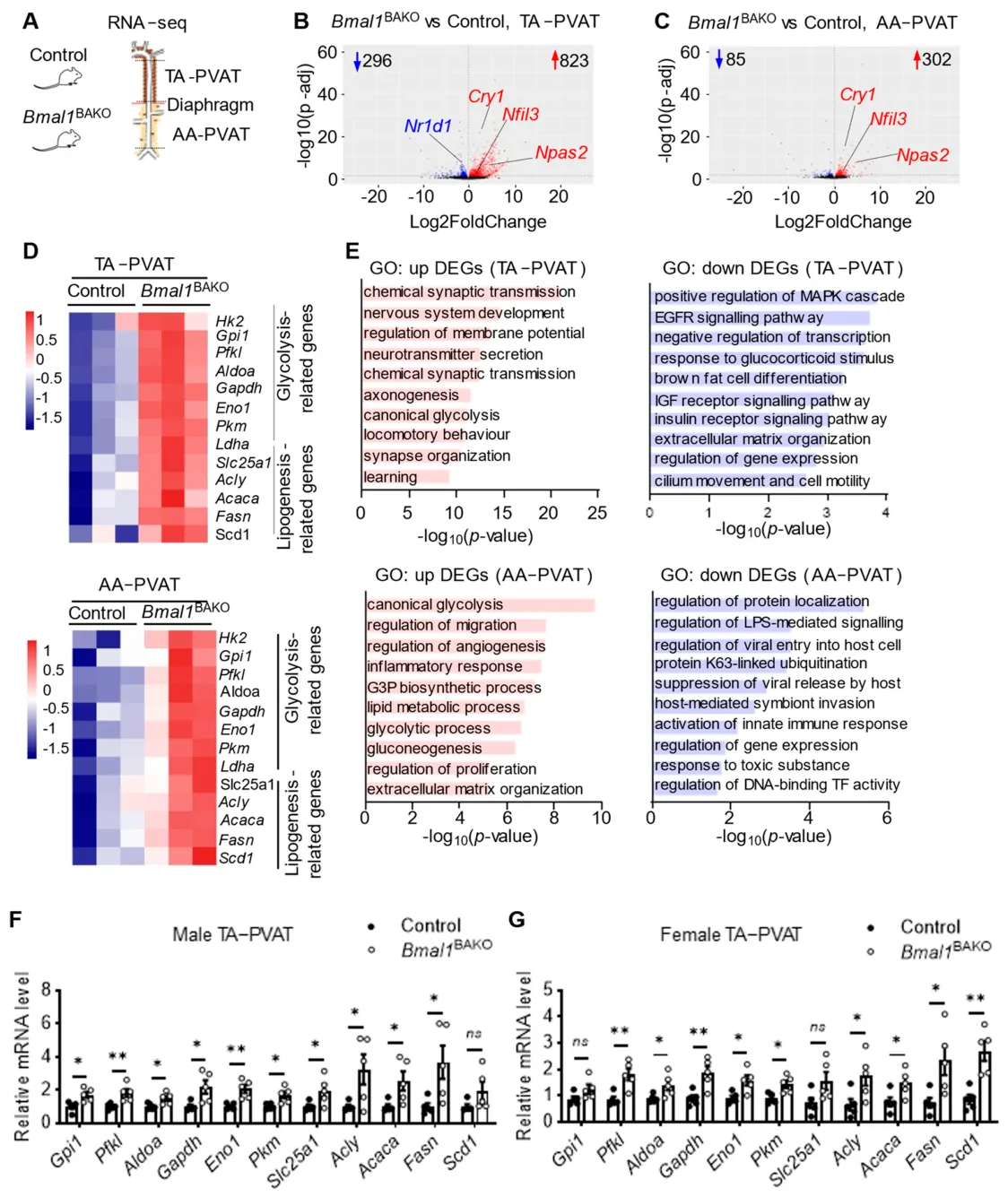
BMAL1 deficiency reprograms PVAT toward a glycolytic and lipogenic transcriptional state. (**A**) Schematic of RNA-seq analysis in thoracic aortic PVAT (TA-PVAT) and abdominal aortic PVAT (AA-PVAT) isolated from control and brown adipocyte specific-*Bmal1* knockout mice (*Bmal1*^BAKO^) (female mice, chow diet, 10–12 weeks of age, *n* = 3 per group). (**B**,**C**) Volcano plots showing differentially expressed genes (DEGs) in TA-PVAT and AA-PVAT. (**D**) Heatmap of representative glycolytic and lipogenic genes showing increased expression in *Bmal1*^BAKO^ PVAT. (**E**) Gene ontology (GO) analysis of upregulated (red) and downregulated (blue) differentially expressed genes in TA-PVAT and AA-PVAT, displaying the top 10 enriched GO terms. (**F**,**G**) qPCR validation of glycolytic and lipogenic gene expression in TA-PVAT from male and female mice. *n* = 5 per group. Data were normalized to *β-actin*. *ns:* no statistical difference. * *p* < 0.05, ** *p* < 0.01 control vs. *Bmal1*^BAKO^ for each gene, by unpaired *t*-test.

To investigate the transcriptional consequences of BMAL1 deletion in PVAT, we utilized a brown adipocyte-selective *Bmal1*-deficient mouse model (*Bmal1*^BAKO^) that was previously established and validated in our prior work, demonstrating efficient depletion of BMAL1 in brown adipocytes [25]. RNA-seq analysis of thoracic aortic PVAT (TA-PVAT) and abdominal aortic PVAT (AA-PVAT) from control and *Bmal1*^BAKO^ mice revealed substantial transcriptional changes in both depots (Figure 1A). The number of differentially expressed genes (DEGs) was greater in TA-PVAT than in AA-PVAT, with 823 upregulated and 296 downregulated genes in TA-PVAT (Figure 1B) and 302 upregulated and 85 downregulated genes in AA-PVAT (Figure 1C). In both depots, BMAL1 loss increased expression of genes involved in glycolysis (*Gpi1*, *Pfkl*, *Aldoa*, *Gapdh*, *Eno1*, *Pkm*) and lipogenesis (*Slc25a1*, *Acly*, *Acaca*, *Fasn*, *Scd1*) (Figure 1D).

Gene ontology analysis showed that upregulated genes in TA-PVAT were enriched for canonical glycolysis and several neural/developmental annotations, whereas downregulated genes were enriched for MAPK signaling, EGFR signaling, insulin and IGF receptor signaling, brown fat cell differentiation, and extracellular matrix organization (Figure 1E). In AA-PVAT, upregulated genes were enriched for canonical glycolysis, G3P biosynthesis, lipid metabolic process, gluconeogenesis, inflammatory response, angiogenesis, and fibroblast proliferation, while downregulated genes were enriched for protein localization, LPS-mediated signaling, viral response pathways, innate immune response, and transcriptional regulation (Figure 1E). qPCR validation in TA-PVAT from male (Figure 1F) and female (Figure 1G) mice confirmed increased expression of glycolytic and lipogenic genes in *Bmal1*^BAKO^ mice.

### 3.2. ChREBP Is Required for BMAL1 Deficiency-Induced Metabolic Gene Expression Changes in Brown Adipocytes

**Figure 2:**
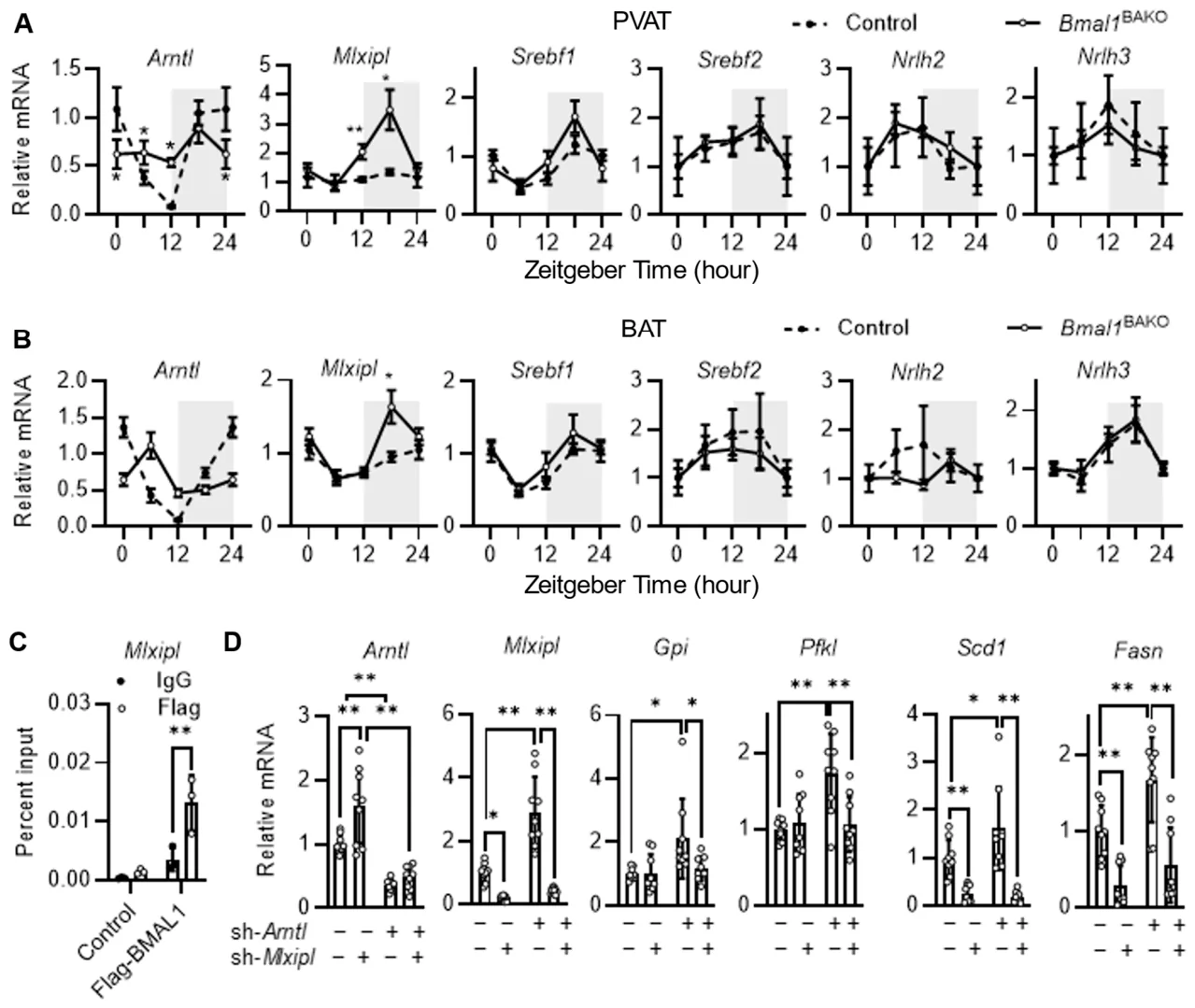
ChREBP contributes to BMAL1 deficiency-induced glycolytic and lipogenic gene programs in brown adipocytes. (**A**,**B**) Time-course quantitative PCR analysis of *Arntl* (encodes BMAL1) and representative metabolic transcription factors and regulators, including *Mlxipl* (encodes ChREBP), *Srebf1*, *Srebf2*, *Nrlh2*, and *Nrlh3* expression in TA-PVAT (**A**) and BAT (**B**) collected from male control and *Bmal1*^BAKO^ mice over a 24-h period (chow diet, 10–12 weeks of age; *n* = 6 per group per ZT point). Gray shading indicates the dark phase (ZT12–ZT24). Data were normalized to *Tbp*. (**C**) Chromatin immunoprecipitation (ChIP) followed by qPCR analysis showing BMAL1 binding to the predicted *Mlxipl* promoter region using the primer sets designed based on the predicted BMAL1-binding sites, in BAT-derived preadipocytes infected with empty vector (control) or Flag-tagged BMAL1 (Flag-BMAL1) (*n* = 3 per group). (**D**) Quantitative PCR analysis of *Arntl*, *Mlxipl*, *Gpi*, *Pfkl*, *Scd1* and *Fasn* expression in primary BAT SVF-derived adipocytes following lentivirus-mediated knockdown of BMAL1 and/or ChREBP (*n* = 9 per group). Data were normalized to Tbp. * *p* < 0.05, ** *p* < 0.01.

Given the glycolytic–lipogenic transcriptional program induced by BMAL1 deletion in PVAT, we next investigated whether altered expression of upstream lipogenic transcription factors contributed to these metabolic gene expression changes. Expression profiles of *Arntl* (encoding BMAL1), *Mlxipl* (encoding ChREBP), *Srebf1* (encoding SREBP-1), *Srebf2* (encoding SREBP-2), *Nr1h3* (encoding LXRα), and *Nr1h2* (encoding LXRβ) were analyzed by qPCR in TA-PVAT (Figure 2A) and BAT (Figure 2B). As expected, *Arntl* expression showed the expected ZT-dependent expression pattern in control mice, whereas its expression was markedly reduced in *Bmal1*^BAKO^ mice across the examined time points. Among the lipogenic transcription factors examined, only *Mlxipl* displayed altered rhythmic expression in TA-PVAT from *Bmal1*^BAKO^ mice, with significantly elevated expression at ZT18, whereas *Srebf1*, *Srebf2*, *Nr1h3*, and *Nr1h2* showed no significant changes. These findings identify selective induction of *Mlxipl*, particularly during the active phase, as a potential mediator of BMAL1 deficiency-induced metabolic gene expression changes in brown adipocytes.

Using the JASPAR database, we identified seven putative BMAL1-binding motifs within the promoter region of *Mlxipl*. To validate these predictions, three pairs of primers were designed targeting distinct E-box-containing regions upstream of the transcription start site (Figure S1A). Chromatin immunoprecipitation (ChIP)-qPCR analysis demonstrated significant enrichment of BMAL1 binding at the region detected by primer set 2, corresponding to the −1737 to −1916 bp region upstream of the transcription start site (Figure 2C). Primer set 1 also showed BMAL1 enrichment (Figure S1B), whereas no significant binding was observed with primer set 3 (Figure S1C).

We next examined whether *Mlxipl* affects the transcriptional effects induced by BMAL1 deficiency. Primary stromal vascular fraction (SVF) isolated from interscapular BAT was subjected to simultaneous knockdown of *Arntl* and *Mlxipl*. Consistent with in vivo findings, *Arntl* knockdown markedly increased the expression of *Mlxipl*, glycolytic genes (*Pfkl*, *Gpi1*), and lipogenic genes (*Fasn*, *Scd1*). Importantly, co-knockdown of *Mlxipl* largely attenuated the *Arntl* knockdown-induced upregulation of these metabolic genes, restoring their expression toward control levels (Figure 2D).

Collectively, these findings support regulation of *Mlxipl* by BMAL1 in brown adipocytes and suggest that loss of BMAL1 contributes to increased *Mlxipl* expression in this cellular context.

### 3.3. Brown Adipocyte BMAL1 Deficiency Does Not Alter Whole-Body Energy Balance While Shifts Adipose Phenotype Toward Lipid Storage

**Figure 3:**
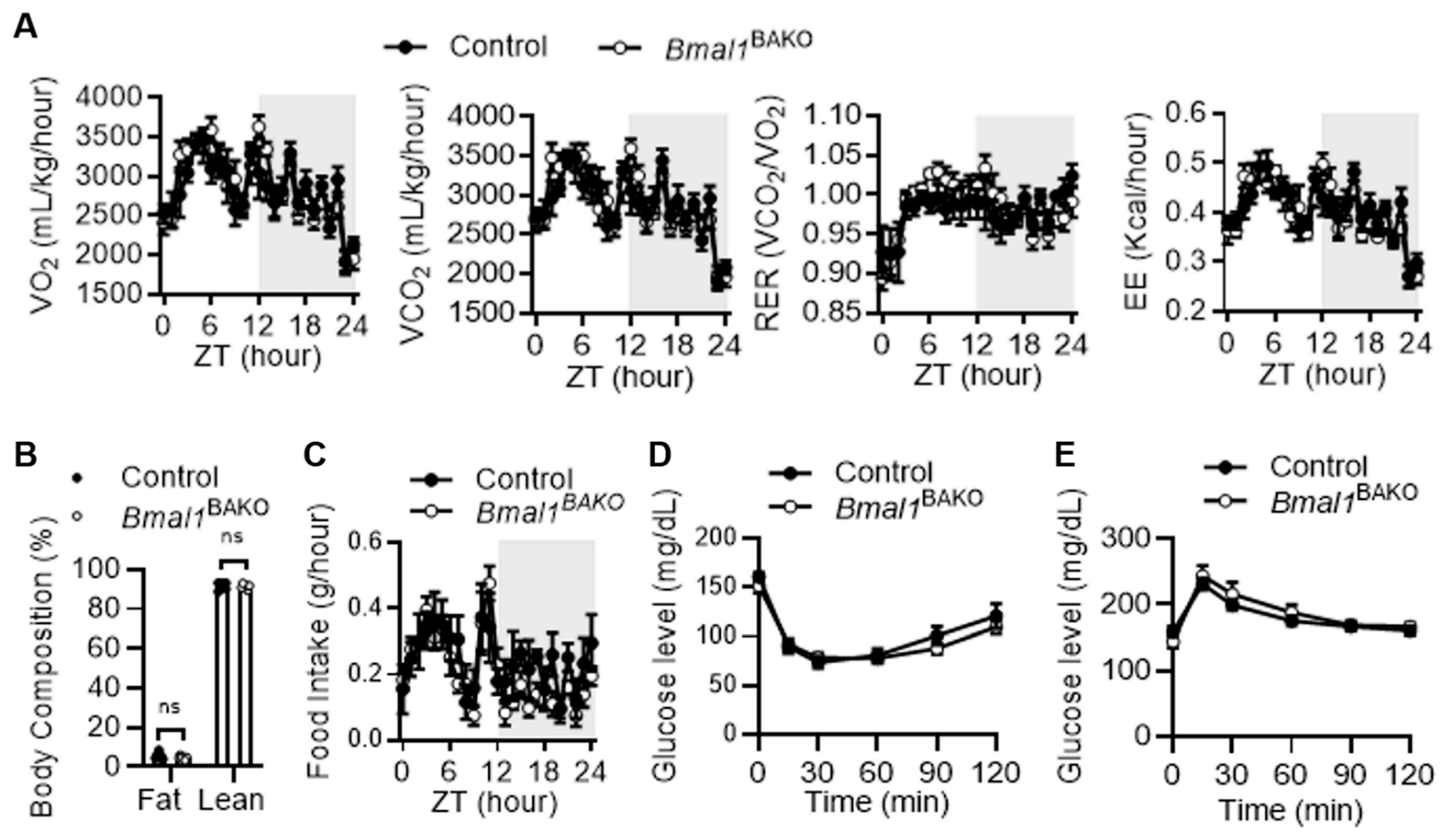
BMAL1 deficiency in brown adipocytes does not alter systemic metabolism under chow diet conditions. (**A**) Metabolic cage measurements including oxygen consumption (VO_2_), carbon dioxide production (VCO_2_) respiratory exchange ratio (RER) and energy expenditure (EE), in male control and *Bmal1*^BAKO^ mice (chow diet, 52 weeks of age; *n* = 6 per group). Gray shading indicates the dark phase (ZT12-ZT24). (**B**) Body composition analysis showing fat mass and lean mass percentages in male mice (chow diet, 52 weeks of age; *n* = 6 per group). (**C**) Average daily food intake measured in metabolic cages, in male control and *Bmal1*^BAKO^ mice (chow diet, 52 weeks of age; *n* = 6 per group). Gray shading indicates the dark phase (ZT12–ZT24). (**D**) insulin tolerance test (ITT) and (**E**) oral glucose tolerance test (OGTT) in male control and *Bmal1*^BAKO^ mice (chow diet, 52 weeks of age; *n* = 7–8 per group). For OGTT, mice were fasted overnight and administered glucose orally (2 g/kg). For ITT, mice were fasted for 6 h and received intraperitoneal insulin (0.75 U/kg). *ns*: no statistical difference.

Aging is associated with altered metabolism [34], and circadian disruption becomes more frequent and pronounced with aging [35]. Next, we assessed whole-body metabolic parameters in 1-year-old *Bmal1*^BAKO^ mice to determine whether BMAL1 deficiency-induced changes in metabolic gene expression in brown adipocytes alter systemic metabolism. Metabolic analyses showed no significant differences in oxygen consumption (VO_2_), carbon dioxide production (VCO_2_), respiratory exchange ratio (RER), or energy expenditure (EE) between *Bmal1*^BAKO^ and control mice (Figure 3A). Body composition was similar between groups, with comparable fat and lean mass (Figure 3B), and daily food intake was unchanged (Figure 3C). Insulin tolerance (Figure 3D) and glucose tolerance (Figure 3E) were also unaffected. Together, these data indicate that BMAL1 deficiency in brown adipocytes does not markedly alter whole-body energy balance or glucose homeostasis.

**Figure 4:**
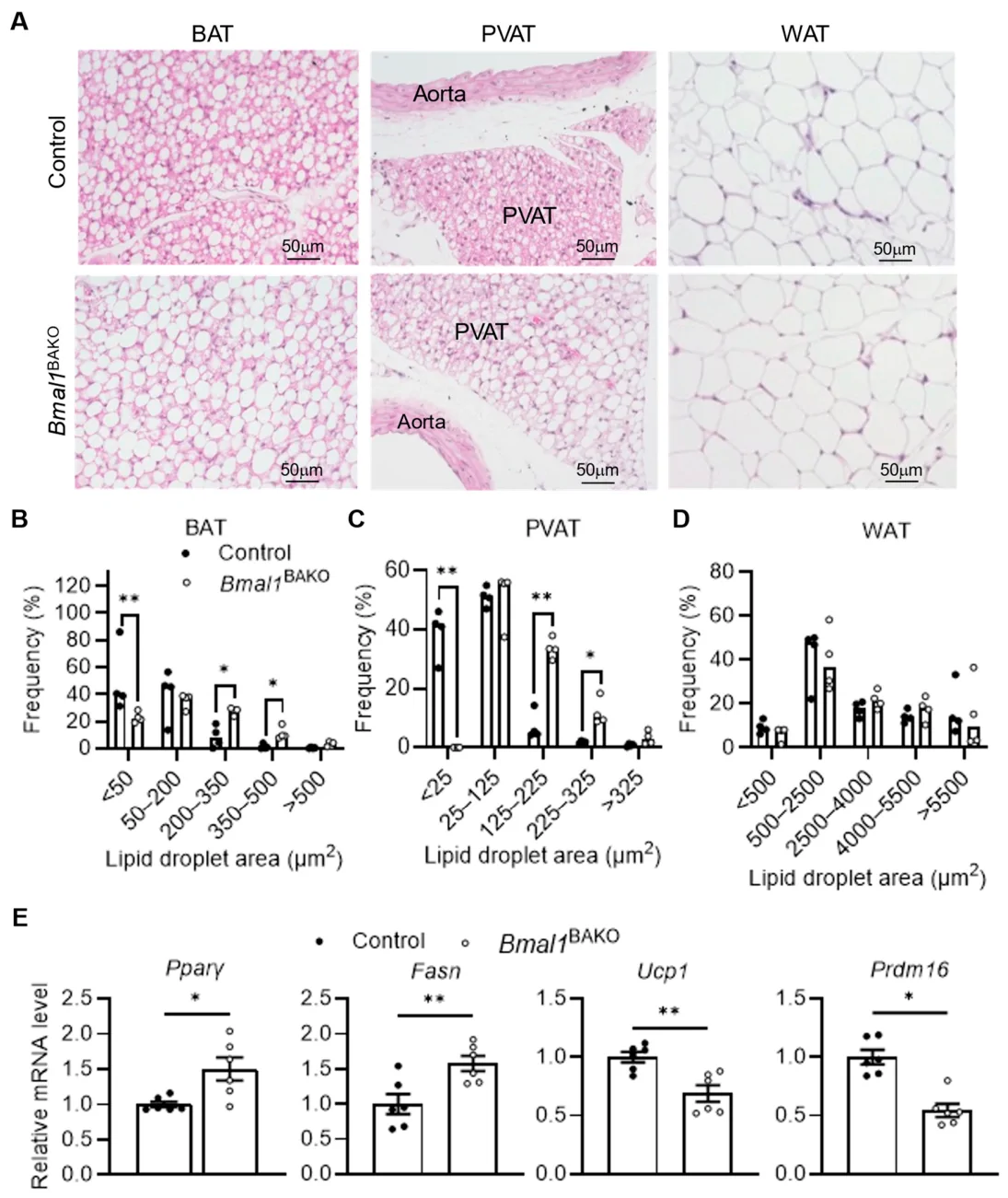
BMAL1 deficiency in brown adipocytes promotes lipid accumulation and PVAT whitening. (**A**) Representative hematoxylin and eosin (H&E) staining images of brown adipose tissue (BAT), perivascular adipose tissue (PVAT) and gonadal white adipose tissue (WAT) from male control and *Bmal1*^BAKO^ mice under chow diet conditions (chow diet, 24 weeks of age, *n* = 4 per group). Scale bars, 100 μm. (**B**–**D**) Quantification of lipid droplet size in BAT (**B**), PVAT (**C**), and WAT (**D**). (**E**) Quantitative PCR analysis of whitening- and browning-related gene expression in PVAT from male control and *Bmal1*^BAKO^ mice (chow diet, 24 weeks of age; *n* = 6 per group), including *Pparγ*, *Fasn*, *Ucp1*, and *Prdm16*. Tissues were harvested at Zeitgeber Time (ZT) 2–3. Data were normalized to β-actin. * *p* < 0.05, ** *p* < 0.01.

Despite no overt systemic metabolic effects, histological analysis of BAT, PVAT, and WAT showed that BMAL1 deficiency altered adipocyte morphology in a depot-specific manner. The most notable change occurred in PVAT and BAT, where lipid droplets were larger and more heterogeneous, consistent with adipose whitening (Figure 4A). Quantification confirmed increased lipid droplet sizes in PVAT and BAT from *Bmal1*^BAKO^ mice, while WAT remained unchanged (Figure 4B–D). These morphologic changes were paralleled by altered marker gene expression: PVAT from *Bmal1*^BAKO^ mice exhibited increased expression of lipogenic genes, including *Pparγ* and *Fasn*, and reduced expression of browning markers such as *Ucp1* and *Prdm16* (Figure 4E), consistent with a shift away from a thermogenic, brown-like state toward lipid storage. Collectively, these data suggest that BMAL1 preserves the brown adipose-like phenotype of PVAT and BAT.

**Figure 5:**
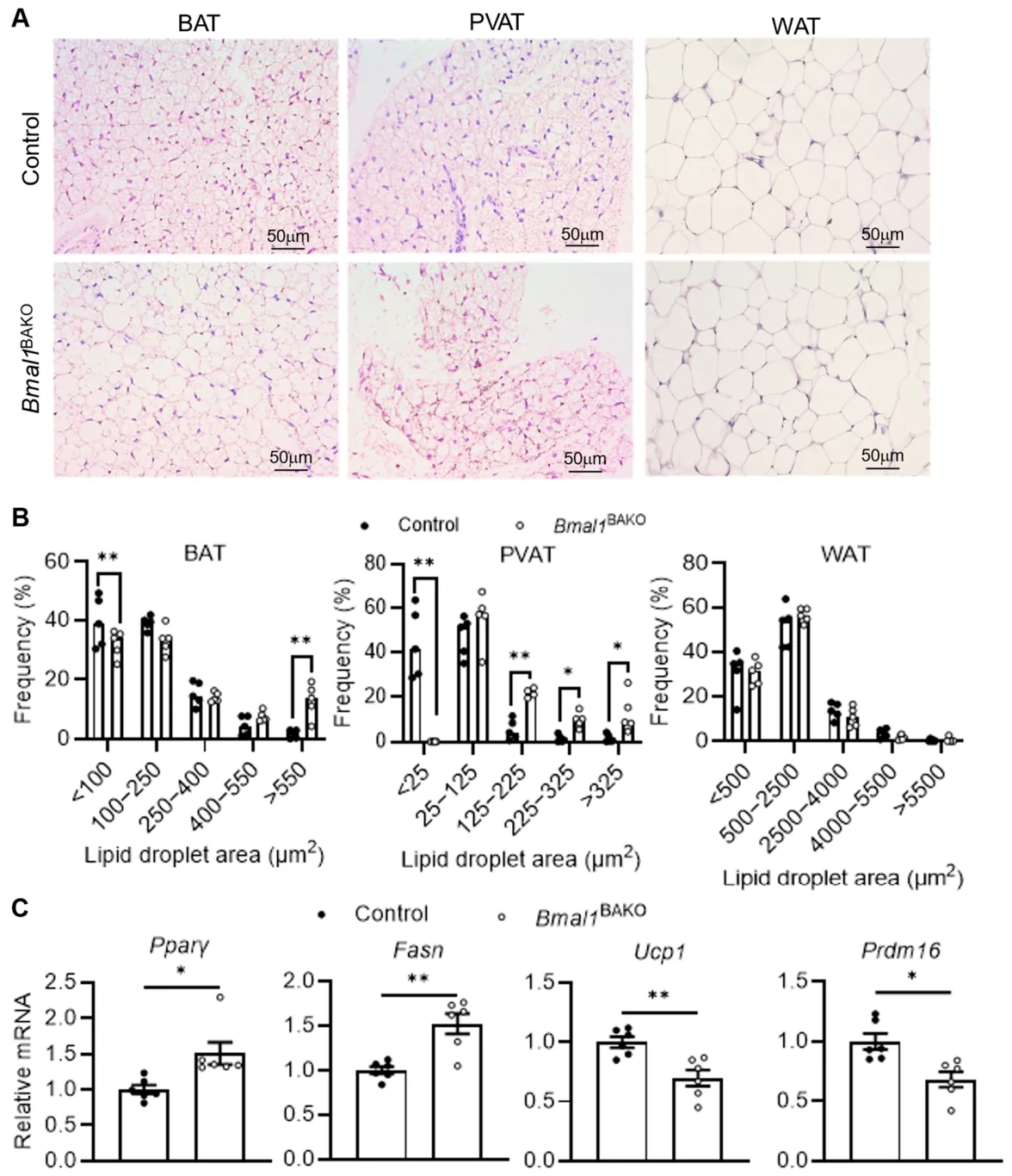
BMAL1 deficiency in brown adipocytes promotes PVAT lipid accumulation and glycolytic-lipogenic gene expression under Western diet. (**A**) Representative hematoxylin and eosin (H&E) staining images of BAT, PVAT and WAT from male control and *Bmal1*^BAKO^ mice (Western diet, 26 weeks of age; *n* = 5 per group). Scale bar, 100 μm. (**B**) Quantification of lipid droplet size in PVAT, BAT and WAT. (**C**) Quantitative PCR analysis of whitening- and browning-related gene expression in PVAT from male control and *Bmal1*^BAKO^ mice (Western diet, 26 weeks of age; *n* = 6 per group), including *Pparγ*, *Fasn*, *Ucp1*, and *Prdm16*. Tissues were harvested at Zeitgeber Time (ZT) 2–3. Data were normalized to β-actin. * *p* < 0.05, ** *p* < 0.01.

**Figure 6:**
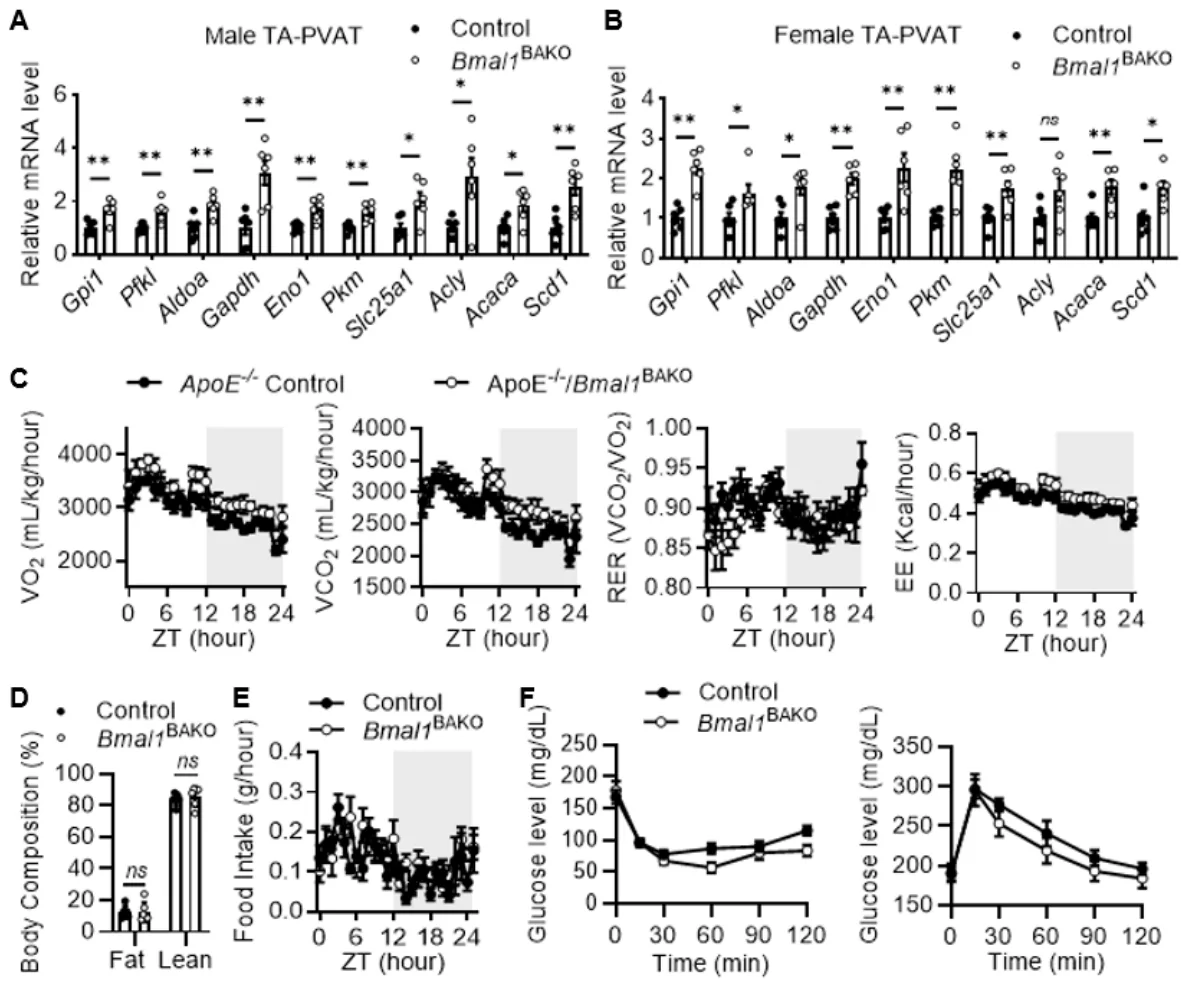
BMAL1 deficiency in brown adipocytes does not alter systemic metabolism under Western diet conditions. (**A**,**B**) Quantitative PCR analysis of representative glycolytic and lipogenic genes in thoracic aortic perivascular adipose tissue (TA-PVAT) from male (**A**) and female (**B**) control and *Bmal1*^BAKO^ mice (Western diet, 26 weeks of age; *n* = 6 per group). Data were normalized to β-actin. (**C**) Metabolic cage measurements including oxygen consumption (VO_2_), carbon dioxide production (VCO_2_), respiratory exchange ratio (RER) and energy expenditure (EE), in male control and *Bmal1*^BAKO^ mice (Western diet, 26 weeks of age; *n* = 6 per group). Gray shading indicates the dark phase (ZT12–ZT24). (**D**) Body composition analysis showing fat mass and lean mass percentages in male control and *Bmal1*^BAKO^ mice (Western diet, 26 weeks of age; *n* = 6 per group). (**E**) Average daily food intake measured in male control and *Bmal1*^BAKO^ mice (Western diet, 26 weeks of age; *n* = 6 per group). Gray shading indicates the dark phase (ZT12–ZT24). (**F**) Insulin tolerance test (ITT) and oral glucose tolerance test (OGTT), in male control and *Bmal1*^BAKO^ mice (Western diet, 26 weeks of age; *n* = 9 control and *n* = 13 *Bmal1*^BAKO^). For OGTT, mice were fasted overnight and administered glucose orally (2 g/kg). For ITT, mice were fasted for 6 h and received intraperitoneal insulin (0.75 U/kg). *ns*: no statistical difference, * *p* < 0.05, ** *p* < 0.01.

We further verified that BMAL1 deficiency in brown adipocytes promotes lipogenesis in young mice after four months of Western diet feeding. Histological analysis revealed increased lipid accumulation in interscapular BAT and TA-PVAT (Figure 5A), with a higher proportion of adipocytes containing enlarged lipid droplets, whereas WAT showed no obvious morphological differences between genotypes (Figure 5B). Under Western diet conditions, BMAL1 deficiency increased lipogenic gene expression, including *Pparγ* and *Fasn*, and reduced thermogenic/browning markers such as *Ucp1* and *Prdm16* in PVAT (Figure 5C). Consistently, key glycolytic and lipogenic genes were broadly upregulated in TA-PVAT from both male and female mice with BMAL1 deficiency (Figure 6A,B), indicating persistent PVAT metabolic reprogramming. However, VO_2_, VCO_2_, RER, EE, body composition, food intake, insulin sensitivity, and glucose tolerance were not significantly different between genotypes (Figure 6C–F). These findings suggest that BMAL1 deficiency in brown adipocytes has limited effects on whole-body energy balance and glucose homeostasis under Western diet feeding conditions.

### 3.4. Brown Adipocyte BMAL1 Deficiency Promotes a Pro-Oxidative and Inflammatory Transcriptional Profile in PVAT and Is Associated with Increased Atherosclerosis

**Figure 7:**
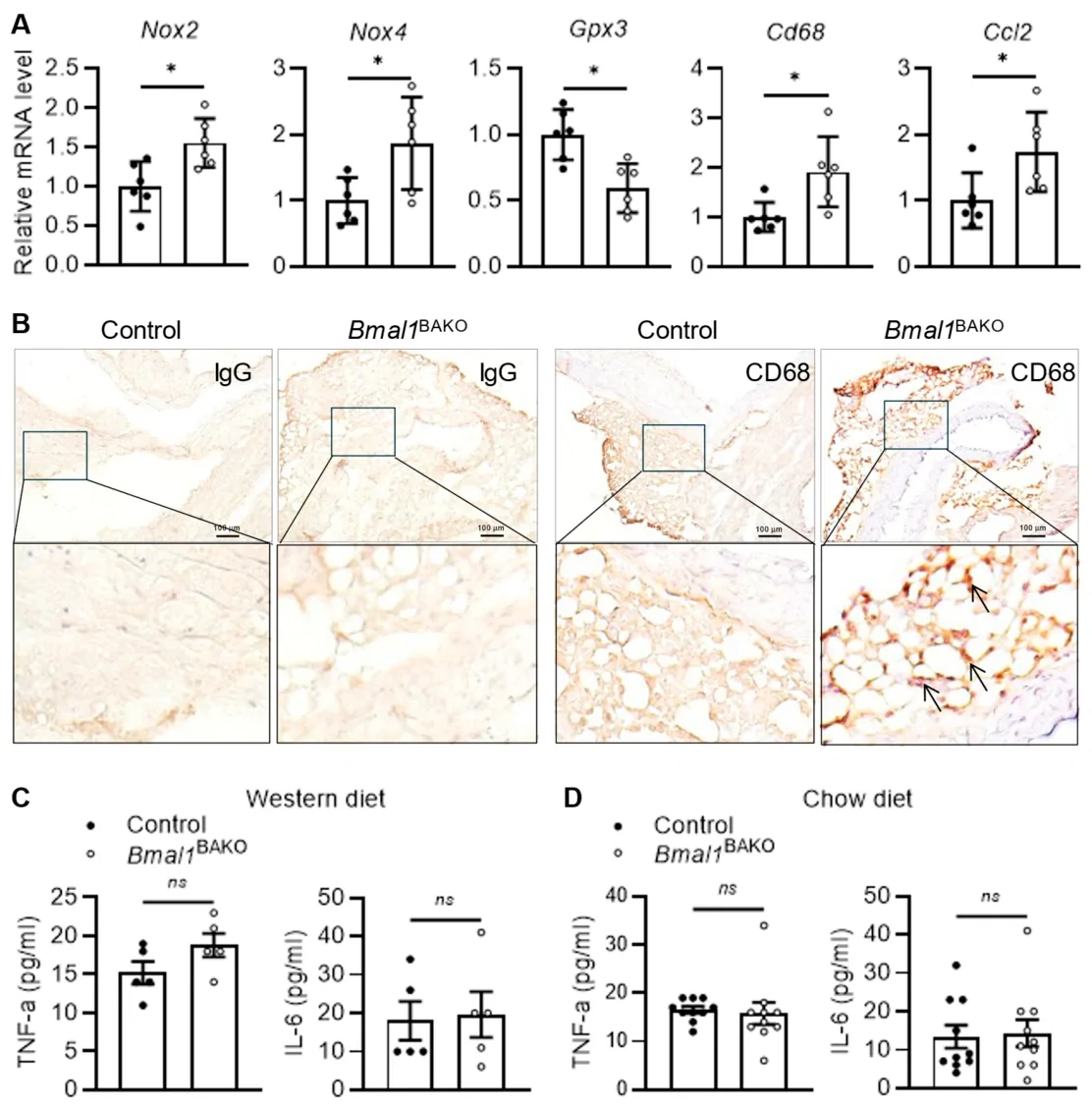
BMAL1 deficiency in brown adipocytes induces oxidative stress-associated gene profiles and inflammatory remodeling in PVAT. (**A**) Quantitative PCR analysis of pro-oxidative and inflammatory gene expression in TA-PVAT from male control and *Bmal1*^BAKO^ mice, including *Nox2*, *Nox4*, *Gpx3*, *Cd68* and *Ccl2* (chow diet, 52 weeks of age; *n* = 6 per group). Tissues were harvested at Zeitgeber Time (ZT) 2–3. Data were normalized to β-actin. (**B**) Representative images showing qualitative assessment of *CD68* and *IgG* in TA-PVAT and the adjacent vascular wall from male control and *Bmal1*^BAKO^ mice (chow diet, 52 weeks of age; *n* = 4 per group). Scale bar, 100 μm. (**C**) Interleukin-6 (IL-6), and tumor necrosis factor α (TNF-α) levels in male mice (Western diet, 26 weeks of age; *n* = 5 control, *n* = 5 *Bmal1*^BAKO^). (**D**) IL-6 and TNF-α levels in male mice (chow diet, 52 weeks of age; *n* = 10 control, *n* = 10 *Bmal1*^BAKO^). *ns*: no statistical difference, * *p* < 0.05.

Consistent with the whitening phenotype observed in PVAT, *Bmal1*^BAKO^ mice exhibited marked local inflammatory activation, accompanied by increased expression of oxidative stress- and immune-related markers. *Nox2* and *Nox4* expression increased and *Gpx3* expression decreased, indicating a shift toward oxidative stress-related gene expression. *Cd68* and *Ccl2* were also upregulated (Figure 7A). Consistently, immunostaining revealed qualitatively more prominent CD68-positive staining in PVAT from BMAL1-deficient mice (Figure 7B). In contrast, circulating TNF-α and IL-6 were not significantly altered (Figure 7C,D), suggesting that the inflammatory response was largely localized rather than systemic. These findings are consistent with a potential contribution of PVAT-localized inflammation to the atherosclerotic phenotype associated with BMAL1 deficiency in UCP1-expressing adipose tissue.

**Figure 8:**
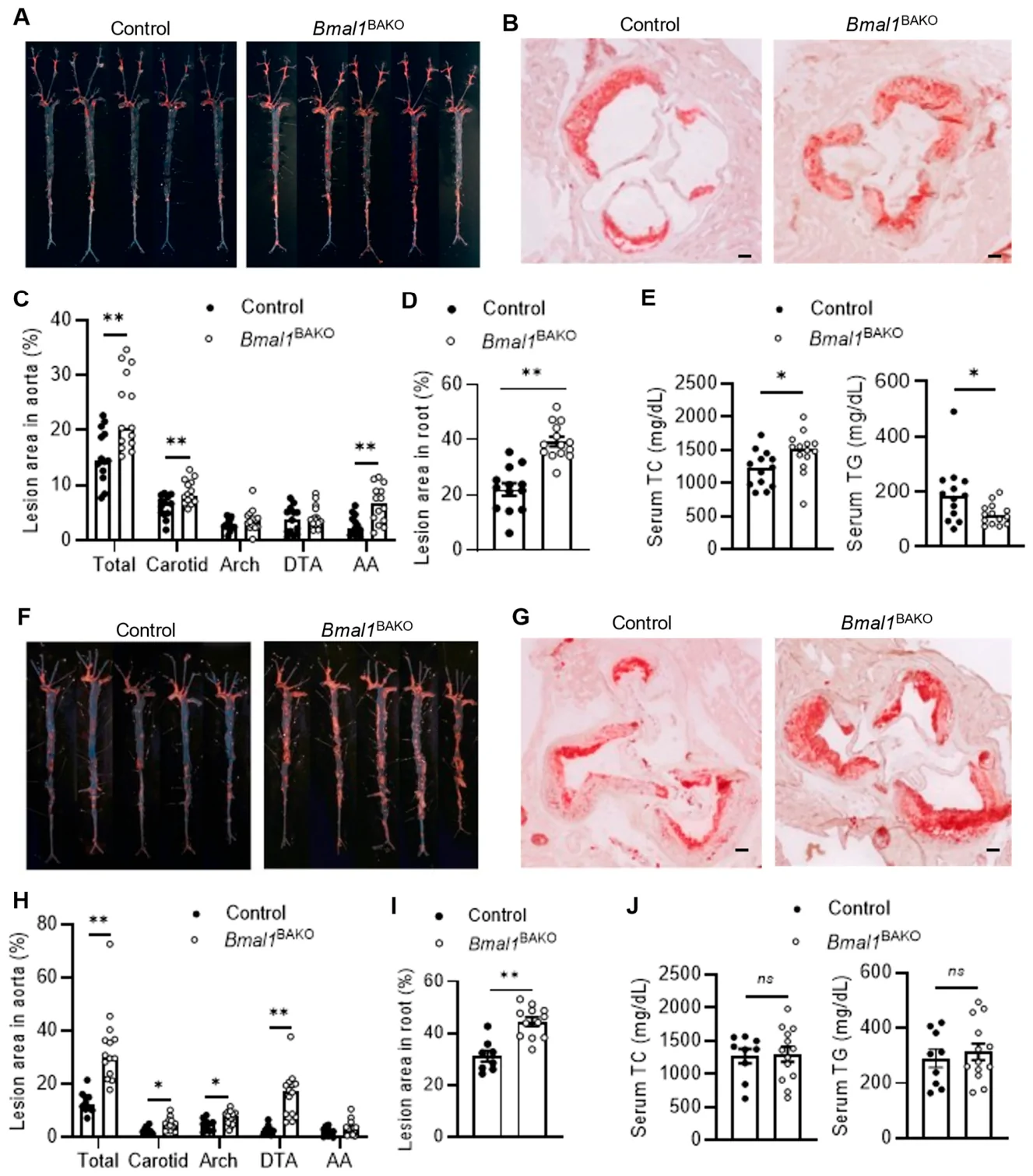
BMAL1 deficiency in brown adipocytes exacerbates atherosclerosis. (**A**) Representative en face Oil Red O-stained aortic trees of male mice following long-term chow diet feeding (chow, diet, 52 weeks of age; *n* = 13 control, *n* = 14 *Bmal1*^BAKO^). (**B**) Representative Oil Red O-stained cross-sections of the aortic sinus of male mice (chow diet, 52 weeks of age; *n* = 13 control, *n* = 14 *Bmal1*^BAKO^). Scale bar, 100 μm. (**C**) Corresponding quantification of en face Oil Red O-stained atherosclerotic lesion area (%) in the carotid arteries (Carotid), aortic arch (Arch), descending thoracic aorta (DTA), and abdominal aorta (AA) of male mice (chow diet, 52 weeks of age; *n* = 13 control, *n* = 14 *Bmal1*^BAKO^). (**D**) Corresponding quantification of lesion area in the aortic sinus of male mice (chow diet, 52 weeks of age; *n* = 13 control, *n* = 14 *Bmal1*^BAKO^). (**E**) Serum total cholesterol (TC) and triglyceride (TG) levels in male mice (chow diet, 52 weeks of age; *n* = 13 control, *n* = 14 *Bmal1*^BAKO^). (**F**) Representative en face Oil Red O-stained aortic trees of male mice following Western diet feeding (Western diet, 26 weeks of age; *n* = 10 control, *n* = 14 *Bmal1*^BAKO^). (**G**) Representative Oil Red O-stained cross-sections of the aortic sinus of male mice (Western diet, 26 weeks of age; *n* = 8 control, *n* = 12 *Bmal1*^BAKO^). Scale bar, 100 μm. (**H**) Corresponding quantification of atherosclerotic lesion area (%) in the Carotid, Arch, DTA, and AA of male mice (Western diet, 26 weeks of age; *n* = 10 control, *n* = 14 *Bmal1*^BAKO^). (**I**) Corresponding quantification of lesion area in the aortic sinus of male mice (Western diet, 26 weeks of age; *n* = 8 control, n = 12 *Bmal1*^BAKO^). (**J**) Serum TC and TG levels in male mice (Western diet, 26 weeks of age; *n* = 9 control, *n* = 11 *Bmal1*^BAKO^). *ns*: no statistical difference, * *p* < 0.05, ** *p* < 0.01.

To determine whether BMAL1 affects atherosclerosis, we assessed plaque burden in aged mice maintained on chow diet for one year and in young mice challenged with Western diet, thereby modeling age-associated and diet-induced atherosclerotic progression. In aged mice on chow diet, en face Oil Red O staining of the entire aortic tree revealed a significantly greater atherosclerotic lesion burden in male *Bmal1*^BAKO^ mice than in controls (Figure 8A). Total lesion area increased from 15.45 ± 1.34% in controls to 23.37 ± 1.86% in *Bmal1*^BAKO^ mice. Quantitative analysis also showed increased plaque area across multiple vascular regions, including the carotid arteries and abdominal aorta (Figure 8C). Aortic sinus sections confirmed expanded plaque areas in male *Bmal1*^BAKO^ mice (22.05 ± 2.24% vs. 39.41 ± 1.71%, *p* < 0.01) (Figure 8B,D). En face analysis also showed increased lesion burden in female *Bmal1*^BAKO^ mice, with total lesion area rising from 13.96 ± 0.86% to 24.06 ± 3.34% (Figure S2A,B). These findings suggest that brown adipocyte BMAL1 deficiency, including in PVAT, promotes atherosclerosis. We next assessed circulating lipid levels. In male mice, serum total cholesterol was slightly increased, and triglycerides were reduced in *Bmal1*^BAKO^ mice (Figure 8E). However, these modest lipid changes were not observed in females and did not parallel the robust increase in lesion burden seen across sexes (Figure S2C).

In young mice fed a Western diet, en face Oil Red O staining of the entire aortic tree revealed a marked increase in atherosclerotic lesion burden in *Bmal1*^BAKO^ mice compared with littermate controls in both sexes. In male mice, total lesion area increased from 13.19 ± 1.27% in controls to 33.68 ± 3.65% in *Bmal1*^BAKO^ mice (*p* < 0.01) (Figure 8F,H). Aortic sinus sections showed a similar increase in lesion area in male mice (31.21 ± 2.15% vs. 44.47 ± 1.74%, *p* < 0.01) (Figure 8G,I). Female mice also showed significantly greater lesion burden, with lesion area increasing from 16.13 ± 1.50% to 30.91 ± 1.82% (*p* < 0.01) (Figure S3A). In male mice, serum total cholesterol and triglycerides were not significantly different between genotypes (Figure 8J). In female mice, total cholesterol was modestly increased in *Bmal1*^BAKO^ mice, whereas triglyceride levels remained comparable between groups (Figure S3C).

Together with the chow-diet findings, these results indicate that BMAL1 deficiency exacerbates atherosclerosis across both aging and dietary contexts.

## 4. Discussion

This study identifies BMAL1 as a key regulator of brown adipocyte biology and atherosclerosis. BMAL1 maintains PVAT homeostasis by restraining a ChREBP-centered glycolytic and lipogenic program. Loss of BMAL1 leads to adipose whitening, oxidative stress, macrophage infiltration, and increased atherosclerotic lesion formation. These findings support a potential role for BMAL1 in linking brown adipocyte dysfunction with cardiovascular pathology. Consistent with our prior work demonstrating that PVAT BMAL1 regulates vascular function through control of angiotensinogen expression and blood pressure [25], the present study expands the role of the brown adipocyte BMAL1 from vascular tone to structural vascular disease.

Emerging evidence from classical BAT depots further supports a critical role for BMAL1 in adipose metabolic regulation. Brown adipocyte-specific deletion of BMAL1 disrupts fatty acid utilization and reduces thermogenic efficiency and whole-body energy expenditure, thereby increasing susceptibility to diet-induced obesity [24]. Mechanistically, loss of BMAL1 in brown adipocytes impairs lipolysis and mitochondrial oxidative capacity, highlighting a requirement for BMAL1 in coordinating substrate mobilization with thermogenic function [36]. In contrast, BMAL1 has also been shown to suppress brown adipogenesis via regulation of TGF-β/BMP signaling, indicating a complex and stage-dependent role in adipose biology [37]. Together, these studies suggest that BMAL1 is not simply a determinant of brown adipocyte identity or thermogenic gene expression, but a central regulator of metabolic efficiency and substrate handling. Our findings extend this paradigm to PVAT, where disruption of BMAL1 shifts adipose metabolism toward a glycolytic-lipogenic and pro-inflammatory state that promotes vascular pathology. The RNA-seq data revealed a strong and coherent shift toward glycolysis and lipogenesis in BMAL1-deficient PVAT. The qPCR validation, gene ontology results, and adipose morphology all pointed in the same direction. This coherence makes the conclusion robust: BMAL1 is not merely a clock gene in PVAT, but a transcriptional regulator of depot identity and metabolic state.

ChREBP is a well-established glucose-responsive transcription factor that controls glycolysis and lipogenesis [38,39]. Its upregulation in *Bmal1*^BAKO^ PVAT, together with BMAL1 occupancy at the *Chrebp* locus and suppression of the metabolic program by ChREBP knockdown, suggests a mechanistic model in which BMAL1 regulates ChREBP-driven transcription. In this way, BMAL1 prevents glucose-derived carbon from being diverted into lipid synthesis within PVAT, thereby preserving a more thermogenic and less inflammatory phenotype.

These findings also help clarify the role of PVAT in vascular disease. PVAT is increasingly viewed as a biologically active tissue that can either protect or harm the vessel wall depending on its state [6]. Brown-like PVAT is generally associated with vascular support [14], whereas white-like or inflamed PVAT is linked to atherosclerosis and vascular dysfunction [13]. The current data show that BMAL1 is required to maintain the protective version of PVAT. When BMAL1 is lost, the tissue adopts a lipogenic [24] (Figure 4A) and inflammatory phenotype (Figure 7A,B) that likely amplifies local plaque-promoting signals under both aging/chow and Western diet conditions (Figure 8A–D,F–I). The stronger effect under Western diet is expected, since lipid stress probably magnifies the consequences of an already dysfunctional PVAT state. However, the fact that lesion burden also increased on chow indicates that the BMAL1-PVAT axis contributes to baseline vascular protection during aging. A key conceptual point is that the vascular phenotype appears to be driven by local dysfunction of PVAT and aortic tissue rather than systemic metabolic collapse. From a mechanistic perspective, the data suggests that BMAL1 maintains a more thermogenic and less lipogenic adipocyte state. In the absence of BMAL1, PVAT shows increased *Pparγ* and *Fasn* and decreased *Ucp1* and *Prdm16*, consistent with a shift away from brown adipocyte identity. At the same time, the tissue becomes enriched for macrophages and oxidative stress markers. This combination of adipocyte whitening and inflammation is a classic recipe for adipose dysfunction and likely underlies the increased atherogenicity of the local microenvironment. Notably, the marked increase in atherosclerotic lesion burden was not accompanied by comparably large alterations in major systemic metabolic parameters. Although changes in selected serum lipid parameters were observed in some experimental groups, the overall metabolic phenotype was relatively limited compared with the pronounced remodeling and inflammatory changes detected in adipose tissue. Together, these findings suggest that the vascular phenotype is largely dissociated from major systemic metabolic disturbances and are consistent with a potential contribution of local adipose–vascular interactions. However, given the non-PVAT specificity of the Ucp1-Cre model and the observed changes in selected circulating parameters, a contribution from systemic or extra-PVAT effects cannot be excluded. 

Several limitations should be acknowledged. First, although the evidence supports BMAL1 as an upstream regulator of ChREBP, the underlying molecular mechanism requires further investigation. ChREBP knockdown attenuated the metabolic transcriptional response to BMAL1 depletion in cultured adipocytes, supporting its role in this in vitro response. However, because ChREBP was not manipulated in vivo, these experiments do not establish that it mediates PVAT remodeling or increased atherosclerotic plaque formation. Second, Ucp1-Cre–mediated Bmal1 deletion is not restricted to PVAT and may also affect other UCP1-expressing depots, including classical BAT. Thus, although the local PVAT changes and their proximity to the vasculature support a PVAT contribution to the atherosclerotic phenotype, contributions from other UCP1-expressing adipose tissues cannot be excluded. Our findings should therefore be interpreted as an association between BMAL1 deficiency in UCP1-expressing adipose tissue, PVAT remodeling, and atherosclerosis, rather than establishing PVAT as the sole mediator. Even so, the consistency of the phenotype across diets, ages, and assays provides strong support for the central conclusions. In conclusion, BMAL1 is an important regulator of metabolic and inflammatory homeostasis in brown adipocytes. BMAL1 deficiency was associated with increased ChREBP-linked glycolytic and lipogenic gene expression, loss of brown-like adipose features, and oxidative stress- and macrophage-related remodeling. In parallel, loss of BMAL1 in brown adipocytes was associated with increased atherosclerosis. Together, these findings suggest that BMAL1 deficiency in UCP1-expressing adipose tissue is associated with PVAT remodeling, local inflammatory activation, and increased atherosclerotic lesion burden. The relatively limited changes in major systemic metabolic parameters, together with the pronounced local adipose tissue phenotype, are consistent with a mechanism that is largely dissociated from major systemic metabolic disturbances. The BMAL1–ChREBP relationship identified in brown adipocytes may contribute to this phenotype, although its causal contribution to PVAT remodeling and atherosclerosis in vivo remains to be established. Because Ucp1-Cre is not restricted to PVAT, contributions from other UCP1-expressing adipose depots cannot be excluded.

## Data Availability

Raw and processed RNA-seq data have been deposited in the National Center for Biotechnology Information Gene Expression Omnibus under accession GSE347651 and will be publicly released no later than publication.

## Supplementary Materials

The following supporting information can be downloaded at: https://www.mdpi.com/article/10.3390/cells15191791/s1, Figure S1: Predicted BMAL1-binding motifs in the *Mlxipl* promoter and ChIP-qPCR primer design; Figure S2. BMAL1 Deficiency in PVAT Exacerbates Atherosclerosis in Female Mice Fed a Chow Diet; Figure S3: BMAL1 Deficiency in PVAT Exacerbates Atherosclerosis in Female Mice Fed a Western Diet; Table S1: Primers used in the study.
