# Supplementary material for: Brown Adipocyte BMAL1 Deficiency Is Associated with PVAT Remodeling and Increased Atherosclerosis
Source: Cells. 2026 Sep 30;15(19):1791. doi: 10.3390/cells15191791 (PMC13664416; doi:10.3390/cells15191791)
Supplement: Supplementary file 1 [file cells-15-01791-s001.zip › cells-4567331-supplementary.pdf]

BMAL1 Deficiency in PVAT Disrupts Metabolic Gene Programs and Aggravates Atherosclerosis

Ruiyan Xu [1,2,†], Zhenguo Wang [2,†], Wenjuan Mu [2], Juan Zhong [2], Yao Liu [1,2], Yanhong Guo [2], Jifeng Zhang [2], Y Eugene Chen [2,*], Guohua Li [1,*], Lin Chang [2,*]

1 Institute of Cardiovascular Disease, Key Laboratory for Arteriosclerology of Hunan Province, Hunan International Scientific and Technological Cooperation Base of Arteriosclerotic Disease, Department of Pathophysiology, Hengyang Medical School, University of South China, Hengyang, Hunan, China.
2 Department of Internal Medicine, Frankel Cardiovascular Center, University of Michigan, Ann Arbor, Michigan, USA.

† these authors contributed equally
* correspondence to Guohua Li: ghli@usc.edu.cn; and Lin Chang: lincha@umich.edu

Supplemental Figure.

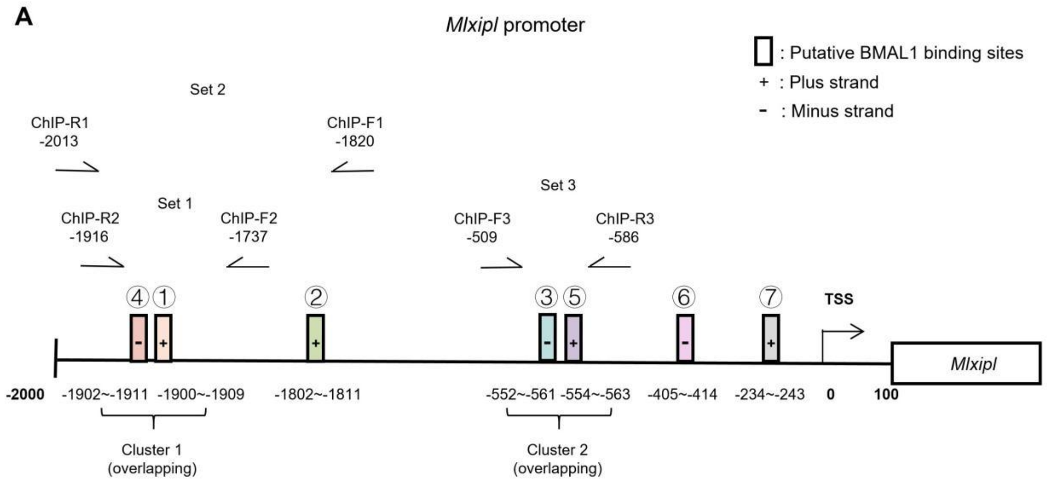

Site ID	Score	Relative score	Start	End	Strand	Predicted sequence
1	9.757898	0.8945768	1902	1911	-	TAGCACGTGG
2	8.293128	0.86128247	1802	1811	+	GGGCACGAGC
3	7.970995	0.85396034	554	563	-	GGGCACATGC
4	7.627058	0.8461427	1900	1909	+	ATCCACGTGC
5	7.12259	0.834676	552	561	+	TGGCATGTGC
6	6.1515913	0.8126052	405	414	-	TCTCAGGTGT
7	5.6402135	0.8009815	234	243	+	AGTCATGAGC

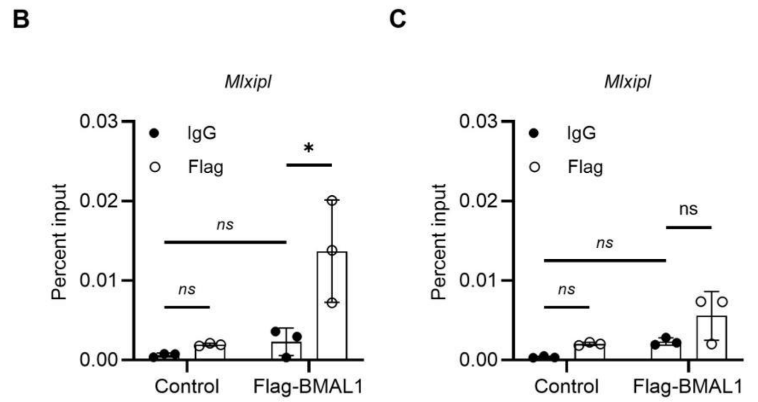

Figure S1. Predicted BMAL1-binding motifs in the *Mlxipl* promoter and ChIP-qPCR primer design. (A) Seven candidate motifs, including overlapping sites are shown relative to the transcription start site (TSS). Three ChIP-qPCR primer sets 1–3 were designed to cover the predicted binding regions for validation of BMAL1 occupancy. Putative BMAL1-binding sites in the mouse *Mlxipl* (encodes *ChREBP*) promoter were identified using JASPAR. **(B-C)** ChIP–qPCR validation of BMAL1 occupancy at predicted binding regions within the *Mlxipl* (encodes *ChREBP*) promoter using primer sets 1 **(B)** and 3 **(C)** in BAT-derived preadipocytes infected with empty vector (control) or Flag-tagged BMAL1 (Flag-BMAL1) (n = 3 per group).

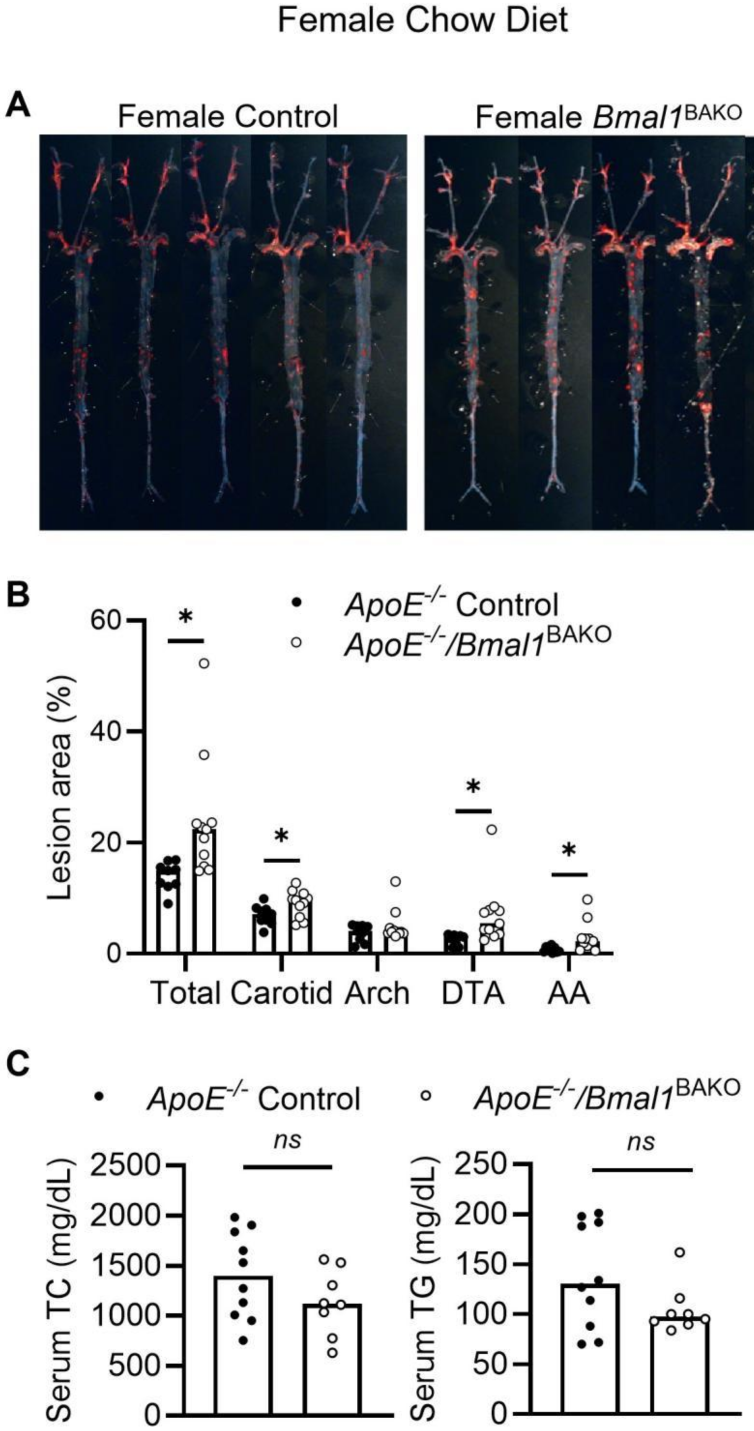

Supplementary Figure S2. BMAL1 Deficiency in PVAT Exacerbates Atherosclerosis in Female Mice Fed a Chow Diet. (A) Representative en face Oil Red O–stained aortic trees of female mice following long-term chow diet feeding (52 weeks of age; n = 9 *Apoe*$^{-/-}$ control, n = 11 *Apoe*$^{-/-}$/*Bmal1*^{BAKO}). **(B)** Corresponding quantification of atherosclerotic lesion area (%) in the Carotid, Arch, DTA, and AA of female mice (chow diet, 52 weeks of age; n = 9 *Apoe*$^{-/-}$ control, n = 11 *Apoe*$^{-/-}$/*Bmal1*^{BAKO}). **(C)** Serum TC and TG levels in female mice (chow diet, 52 weeks of age; n = 9 *Apoe*$^{-/-}$ control, n = 11 *Apoe*$^{-/-}$/*Bmal1*^{BAKO}).

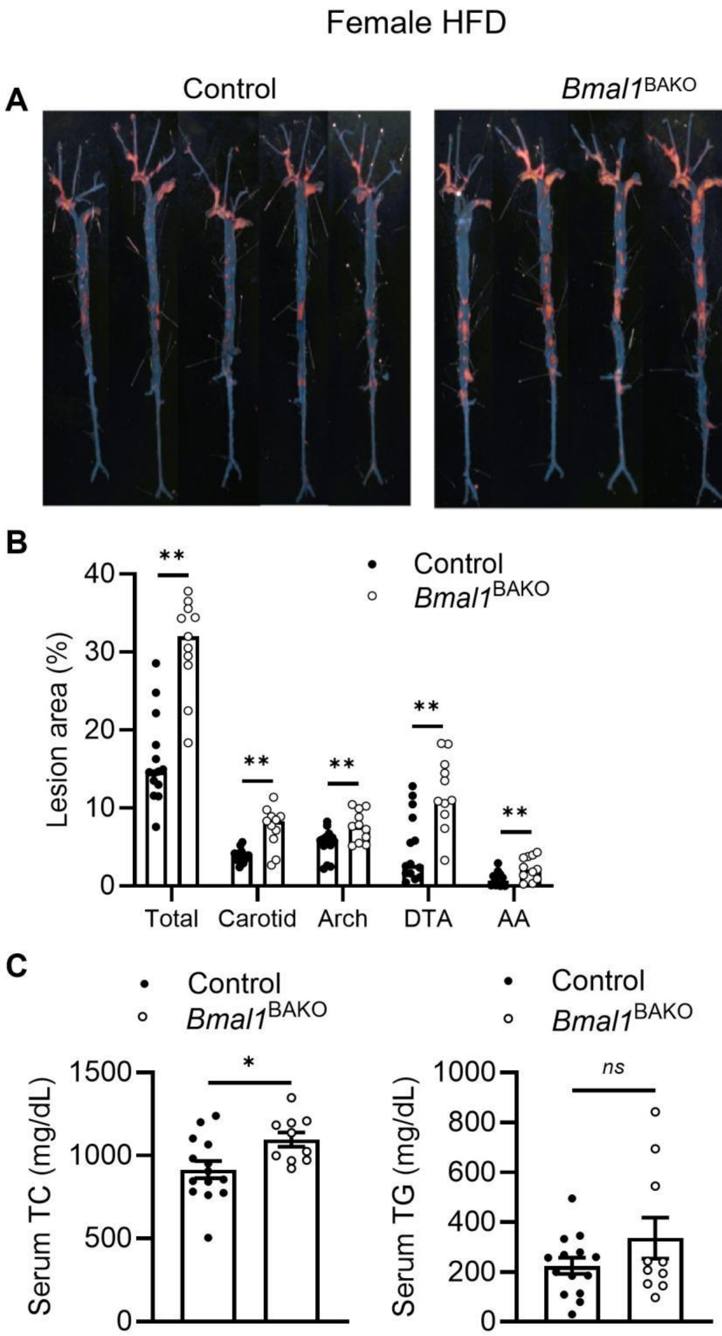

Supplementary Figure S3. BMAL1 Deficiency in PVAT Exacerbates Atherosclerosis in Female Mice Fed a Western Diet. (A) Representative en face Oil Red O–stained aortic trees from female *Apoe*$^{-/-}$ control and *Apoe*$^{-/-}$/*Bmal1*^{BAKO} mice following Western diet feeding (Western diet, 26 weeks of age; n = 14 *Apoe*$^{-/-}$ control, n = 10 *Apoe*$^{-/-}$/*Bmal1*^{BAKO}). **(B)** Corresponding quantification of atherosclerotic lesion area (%) (Western diet, 26 weeks of age; n = 14 *Apoe*$^{-/-}$ control, n = 10 *Apoe*$^{-/-}$/ *Bmal1*^{BAKO}). **(C)** TC and TG levels in female mice (Western diet, 26 weeks of age; n = 14 *Apoe*$^{-/-}$control, n = 10 *Apoe*$^{-/-}$/*Bmal1*^{BAKO}).

Supplemental Table S1. Primers used in the study

mRNA	Forward primer	Reverse primer
Tbp	ACCCTTCACCAATGACTCCTATG	TGACTGCAGCAAATCGCTTGG
Bmal1	AGGCCCACAGTCAGATTGAAA	CCAAAGAAGCCAATTCATCAATG
Cry1	TCGCCGGCTCTTCCAA	TCAAGACACTGAAGCAAAAATCG
Per1	TCGAAACCAGGACACCTTCTCT	GGGCACCCCGAAACACA
Gpi1	CCGTGTCTGGTTTGTCTCTAAC	AACCACTCCTTTGCTGTCTC
Pfkl	GGAAAGCCTATCTCATCCAGC	CCATACCCATCTTGCTACTCAG
Aloda	GCCTGAAATTCTCCCTGATGG	GTGCCTTCCAGATAGACATGG
Gapdh	CTGCGACTTCAACAGCAACT	GAGTTGGGATAGGGCCTCTC
Eno1	CAGAATTGAGGAAGAGCTGGG	TGACATCAGGGATAGAGGACC
Pkm	CCATTCTCTACCGTCCTGTTG	TCCATGTAAGCGTTGTCCAG
Slc25a1	CAGAAGCAGTGGTAGTCGTG	TTCCCTTTAGCCCTTGTTCC
Acly	CAGCCAAGGCAATTTCAGAGC	CTCGACGTTTGATTAACTGGTCT
Acaca	AAGGCTATGTGAAGGATGTGG	CTGTCTGAAGAGGTTAGGGAAG
Fasn	GCTGGCATTCGTGATGGAGTCGT	AGGCCACCAGTGATGATGTAACTCT
Scd1	GCGATACACTCTGGTGCTCA	CCCAGGGAAACCAGGATATT
Chrebp	AATGGGATGGTGTCTACCGC	GGCGAAGGGAATTCAGGACA
Srebp1	CAGCCCTCCACCAGGTAATA	GGTTGGGAGTAGAGGGAAGG
Srebp2	CCATCTTCCCCTCTCTTTCC	AGGGAAGATCCTGGGAGAAA
Lxra	TGCCATCAGCATCTTCTCTG	GGCTCACCAGCTTCATTAGC
Lxrb	CGCTACAACCACGAGACAGA	TGTTGATGGCGATAAGCAAG
Chrebp-α	GAAGCCACAGTGAAATCTCG	TTGTTCAGCCGGATCTTGTC
Chrebp-β	TCTGCAGATCGCGTGGAG	CTTGTCCCGGCATAGCAAC
Gpx3	CCTTTTAAGCAGTATGCAGGCA	CAAGCCAAATGGCCCAAGTT
Nox2	TGTGGTTGGGGCTGAATGTC	CTGAGAAAGGAGAGCAGATTTCG
Nox4	CCTTTTACCTATGTGCCGGAC	CATGTGATGTGTAGAGTCTTGCT
Cd68	ACACTTCGGGCCATGTTTCT	GGGGCTGGTAGGTTGATTGT
Ccl2	GGTCTTCAGCACCTTTGAATG	ATTAAGGCATCACAGTCCGAG
Tnfα	CAGGCGGTGCCTATGTCTC	CGATCACCCCGAAGTTCAGTAG
Il1-β	GGGCCTCAAAGGAAAGAATC	TACCAGTTGGGGAACTCTGC
Ppaγ	GCCAGTTTCGATCCGTAGAA	AATCCTTGGCCCTCTGAGAT
Ucp1	CAGCACGGTGAAGCCATTC	GCGTGCATCCGCTTGTG
Prdm16	CTCACTCAGGATTGGCCTCTAC	TCTGACCTTCACGACCTCTGTA
